# Non-invasive Assessment of Lower Limb Geometry and Strength Using Hip Structural Analysis and Peripheral Quantitative Computed Tomography: A Population-Based Comparison

**DOI:** 10.1007/s00223-015-0081-7

**Published:** 2015-11-21

**Authors:** A. E. Litwic, M. Clynes, H. J. Denison, K. A. Jameson, M. H. Edwards, A. A. Sayer, P. Taylor, C. Cooper, E. M. Dennison

**Affiliations:** MRC Lifecourse Epidemiology Unit, (University of Southampton) Southampton General Hospital, Southampton, SO16 6YD UK; NIHR Musculoskeletal Biomedical Research Unit, University of Oxford, Oxford, UK; Victoria University of Wellington, Wellington, New Zealand; Institute for Ageing and Institute of Health & Society, Newcastle University, Callaghan, Australia

**Keywords:** Osteoporosis, Epidemiology, Hip structural analysis, Peripheral quantitative computed tomography

## Abstract

Hip fracture is the most significant complication of osteoporosis in terms of mortality, long-term disability and decreased quality of life. In the recent years, different techniques have been developed to assess lower limb strength and ultimately fracture risk. Here we examine relationships between two measures of lower limb bone geometry and strength; proximal femoral geometry and tibial peripheral quantitative computed tomography. We studied a sample of 431 women and 488 men aged in the range 59–71 years. The hip structural analysis (HSA) programme was employed to measure the structural geometry of the left hip for each DXA scan obtained using a Hologic QDR 4500 instrument while pQCT measurements of the tibia were obtained using a Stratec 2000 instrument in the same population. We observed strong sex differences in proximal femoral geometry at the narrow neck, intertrochanteric and femoral shaft regions. There were significant (*p* < 0.001) associations between pQCT-derived measures of bone geometry (tibial width; endocortical diameter and cortical thickness) and bone strength (strength strain index) with each corresponding HSA variable (all *p* < 0.001) in both men and women. These results demonstrate strong correlations between two different methods of assessment of lower limb bone strength: HSA and pQCT. Validation in prospective cohorts to study associations of each with incident fracture is now indicated.

## Introduction

Hip fracture is the most significant complication of osteoporosis in terms of mortality, long-term disability and decreased quality of life. Approximately 50 % of patients suffering a hip fracture can no longer live independently and 20 % die within 12 months of the fracture [1]. It is also a major public health issue due to health and social costs. In the UK, about 70,000–75,000 hip fractures occur each year. These account for over 20 % of orthopaedic bed occupancy with an annual cost of approximately £2 billion [2]. With rising life expectancy worldwide, the number of elderly individuals is increasing globally and it is estimated that the incidence of hip fracture will rise from 1.66 million in 1990 to 6.26 million by 2050 [3]. Hence techniques that best predict fracture risk are invaluable.

While bone mineral density (BMD) is a well-recognised strong predictor of osteoporotic fracture [4], proximal femur geometry (PFG) parameters have also been proposed to be predictive of mechanical strength and femoral neck fracture risk, as bone shape adjusts the transmission of the impact forces through the bone, contributing to the effective stress within the bone [5]. Previous cadaveric studies suggested that mechanical characteristics of the proximal femur as assessed by measures of femoral geometry such as femoral width and cross-sectional moment of inertia add to information obtained from BMD measurement by DXA [6]. Further work has reported an association between the hip axis length (HAL) measured by DXA scans and hip fracture risk [7]. In subsequent work, PFG parameters including HAL and neck-shaft angle have been reported to predict hip fracture independent of BMD [8, 9].

Peripheral quantitative computed tomography (pQCT) provides additional geometric variables such as the true volumetric BMD (vBMD) and represents a three-dimensional technique of measuring vBMD that is not confounded by bone size. It also yields separate measures of bone strength and geometry of the trabecular and cortical bone. Some studies have suggested that these parameters might provide a more in-depth understanding of bone strength and better fracture prediction beyond areal BMD (aBMD) obtained by DXA [10, 11]; for example, pQCT-derived bone parameters differ among individuals with fracture and those without fracture [11–14]. Further work has reported an association between strength/geometry parameters measured by pQCT with fractures [10, 12, 14]. In subsequent work, it was reported that individuals with fractures had lower or less favourable bone strength/geometry than those without fractures [15]. As a peripheral technique, pQCT offers information on bone structure and strength at an alternative lower limb site to hip structural analysis (HSA), namely the tibia.

To date, however, no study has compared bone strength analyses using these two complementary techniques. Here we consider the relationships between these two methods of assessment of lower limb bone strength (HSA and pQCT).

## Methods

The Hertfordshire Cohort Study (HCS) is a population-based cohort study in the UK which was designed to examine the relationship between growth in infancy and the subsequent risk of adult disease, including osteoporosis. Study design and recruitment have been described in detail previously [8] but in brief we studied 431 women and 488 men, 59–71 years of age, who were born between 1931 and 1939 in Hertfordshire and still lived there at the time of the baseline visit for this study (in 1998–2003). The participants of the HCS are known to be representative of elderly men and women in the UK for lifestyle determinants of bone mass.

After obtaining written permission from each person’s general practitioner, we approached each person by letter, asking them whether they would be willing to be contacted by one of our research nurses. A detailed lifestyle questionnaire was administered to all participants to obtain information regarding medical history and lifestyle including cigarette smoking, alcohol consumption, physical activity, socioeconomic status and, in women, years since menopause and use of oestrogen replacement therapy. Dietary calcium intake was calculated from a food frequency questionnaire. Height was measured to the nearest 0.1 cm using a Harpenden pocket stadiometer, and weight was measured to the nearest 0.1 kg on a SECA floor scale. Body mass index (BMI) was calculated as weight divided by height^2^ (kg/m^2^).

At an initial clinic visit, eligible subjects were invited to book a return visit over the coming weeks for bone density measurements. Participants taking bisphosphonates were excluded from this part of the study, although women taking hormone replacement therapy (HRT) were allowed to participate as a large number of otherwise eligible women were taking this medication. Individuals taking oral glucocorticoids were excluded. BMD was measured in each subject, by dual energy X-ray absorptiometry (DXA) at the lumbar spine and proximal femur using a Hologic QDR 4500 instrument (Vertec Scientific, Reading, UK). Measurement precision error, expressed as coefficient of variation, was 1.55 % for lumbar spine BMD, 1.45 % for total femur and 1.83 % for femoral neck BMD for the Hologic QDR 4500. All geometrical parameters assessed were extracted from scans using standard Hologic software.

The Hologic Hip Structural Analysis programme was employed to measure the structural geometry of the left hip for each scan. The bone mass image is used directly from the DXA scan where pixel values are expressed in areal mass (g/cm^2^). The programme analyses cross-sections traversing the proximal femur at three specific locations: the narrow neck across the narrowest diameter of the femoral neck; the intertrochanteric along the bisector of the neck-shaft angle; and the shaft, 2 cm distal to the midpoint of the lesser trochanter. Each scan was checked for correct placement of the region of interest by a research assistant. Data was exported in spread sheet form for analysis.

pQCT measurements were obtained 4 years after baseline DXA scans at a subsequent clinic visit. A tibial scan (non-dominant side) was performed using a Stratec 2000 instrument. A scout view was performed on the lower leg to identify a baseline for the measurements. The middle of the distal cortical end of the tibia was used as a reference line. Four slices were taken for the lower leg scan (4, 14, 38 and 66 %). Measurement precision error, expressed as a coefficient of variation, was typically around 1–3 %. These figures were obtained by 20 volunteers who were part of the study undergoing 2 scans on the same day, the limb repositioned in the machine between examinations. The threshold for bone was set at 280 mg/cm^3^. Bone strength was estimated with respect to torsion (polar strength strain index or SSI.

Data were analysed using the Stata statistical software package version 13. Study participant characteristics for continuous variables were calculated as means and standard deviations, (SD) or medians and interquartile ranges (IQR) as appropriate. Categorical and binary variables were summarised as numbers and percentages of the total study population. All data were inspected for normality. Variables with a skewed distribution were normalised by an appropriate transformation where necessary. A visual assessment indicated linear relationships between DXA and HSA variables. These relationships were further examined using Pearson’s correlation coefficients. A *p* value of ≤0.05 was considered to be significant for all analyses.

Ethical permission for the study was granted by the East and North Hertfordshire Ethical Committees. All participants gave written informed consent.

## Results

The characteristics of the study population are displayed in Table 1. The mean age of men and women in the study was 64.8 and 66.3 years, respectively. Men were more physically active and reported higher dietary calcium intakes than women. Among men, 66.1 % were current or ex-smokers compared with only 37.6 % of women. Similarly men consumed higher quantities of alcohol than women.

**Table 1:** Summary characteristics of the study participants

Characteristic	Total N	Men Mean (SD)	Total N	Women Mean (SD)	*p* value^a^
Age (years)	488	64.8 (2.5)	431	66.3 (2.6)	<0.001
BMI (kg/m^2^)^b^	488	26.6 (1.1)	431	26.8 (1.2)	0.497
Dietary calcium intake (mg/day)^c^	488	1214 (1.3)	431	1087 (1.3)	<0.001
Activity score	488	64.1 (14.8)	431	61.3 (14.7)	0.004
BMD total hip (g/cm^2^)	488	1.04 (0.13)	431	0.9 (0.13)	<0.001

	Total N	Men Median (IQR)	Total N	Women Median (IQR)	*p* value
Alcohol consumption (units/week)	488	9.5 (2.5–21.6)	431	1.5 (0.0–6.0)	<0.001

	Total *N*	Men *N* (%)	Total *N*	Women *N* (%)	*p* value
Smoking status	488		430		<0.001
Current		71 (14.6)		41 (9.5)	0.517
Ex		252 (51.6)		121 (28.1)	n/a
Never		165 (33.8)		268 (62.3)	n/a
Social class	462		431		0.140
I–IIINM		191 (41.3)		169 (39.2)	
IIIM–V		271 (58.7)		262 (60.8)	
Oestrogen	n/a	n/a	431		n/a
Replacement					
Never				252 (58.5)	
>5 years ago				79 (18.3)	
<5 years ago				23 (5.3)	
Current				77 (17.9)	
Years since	n/a	n/a	428		n/a
Menopause					
0–10				55 (12.9)	
10–20				202 (47.2)	
>20				69 (16.1)	
Hysterectomy				102 (23.8)	
Number of comorbidities^c^	461		412		
0		251 (54.4)		220 (53.4)	
1		139 (30.2)		133 (32.3)	
2		53 (11.5)		53 (12.9)	
3 or more		18 (3.9)		6 (1.5)	

^a^ *p* value for the difference between men and women

^b^Geometric mean

^c^Number of comorbidities out of bronchitis, diabetes, IHD, hypertension and stroke

*BMI* body mass index

In total 576 subjects provided pQCT scans for these analyses, 291 men and 276 women. We observed strongly significant sex differences in most femoral geometry parameters measured (Table 2). In all cases, measures of size and strength were greater in men than women. Buckling ratio was higher in women than men at the narrow neck, intertrochanteric region and femoral shaft (*p* = 0.016, *p* < 0.001 and *p* = 0.001, respectively). Neck-shaft angle was greater in men than women (*p* = 0.034). Table 3 shows correlations between HSA variables and pQCT variables measured in the tibia. There were significant (*p* < 0.001) associations between pQCT- and HSA-derived measures of bone width, endocortical diameter, cortical thickness and bone strength (strength strain index and section modulus). In particular, we observed strong relationships between tibial polar SSI at the 38 % slice with narrow neck section modulus (*r* 0.40; *p* < 0.001 in men); intertrochanteric section modulus (*r* 0.46; *p* < 0.001 in men) and femoral shaft section modulus (*r* 0.54; *p* < 0.001 in men) highlighting strong relationships between measures of strength assessed using both techniques. Strong relationships were also observed between pQCT and HAS bone geometry. For example, tibial cortical thickness at the 38 % slice with narrow neck cortical thickness (*r* 0.39; *p* < 0.001 in men); intertrochanteric cortical thickness (*r* 0.46; *p* < 0.001 in men) and femur shaft cortical thickness (*r* 0.52; *p* < 0.001 in men). In women, the correlation coefficient was numerically higher than in men: tibial cortical thickness at the 38 % slice with narrow neck cortical thickness (*r* 0.49; *p* < 0.001); intertrochanteric cortical thickness (*r* 0.61; *p* < 0.001) and femur shaft cortical thickness (*r* 0.63; *p* < 0.001). However, no formal statistical assessment of the strength of correlation by sex was undertaken.

**Table 2:** Summary of femoral geometry parameters assessed by DXA

	Men (*n* = 488)	Women (*n* = 431)	*p* value^a^
Narrow neck			
CSMI (cm^4^)	4.4 (1.0)	2.6 (0.7)	<0.001
Width (cm)	3.8 (0.2)	3.3 (0.3)	<0.001
ED (cm)	3.4 (0.2)	3.0 (0.3)	<0.001
ACT (cm)	0.2 (0.0)	0.2 (0.0)	<0.001
PCD (cm)	1.7 (0.1)	1.5 (0.2)	<0.001
CMP	0.4 (0.0)	0.4 (0.0)	<0.001
Section modulus (cm^3^)	2.1 (0.4)	1.4 (0.3)	<0.001
Buckling ratio	11.1 (2.3)	11.5 (3.0)	0.016
Intertrochanter			
CSMI (cm^4^)	25.2 (6.1)	15.3 (3.8)	<0.001
Width (cm)	6.4 (0.4)	5.7 (0.4)	<0.001
ED (cm)	5.4 (0.4)	4.8 (0.5)	<0.001
ACT (cm)	0.5 (0.1)	0.4 (0.1)	<0.001
PCD (cm)	2.9 (0.2)	2.5 (0.3)	<0.001
CMP	0.4 (0.0)	0.4 (0.0)	<0.001
Section modulus (cm^3^)	7.1 (1.4)	4.8 (1.0)	<0.001
Buckling ratio	7.4 (1.4)	7.9 (1.7)	<0.001
Femur shaft			
CSMI (cm^4^)	6.0 (1.4)	3.6 (0.9)	<0.001
Width (cm)	3.3 (0.2)	3.0 (0.2)	<0.001
ED (cm)	2.0 (0.4)	1.8 (0.4)	<0.001
ACT (cm)	0.7 (0.1)	0.6 (0.1)	<0.001
PCD (cm)	1.6 (0.1)	1.5 (0.1)	<0.001
CMP	0.5 (0.0)	0.5 (0.0)	<0.001
Section modulus (cm^3^)	3.4 (0.6)	2.4 (0.4)	<0.001
Buckling ratio	2.7 (0.6)	2.9 (0.8)	0.001
Neck-shaft angle (degrees)	129.5 (5.5)	128.7 (5.3)	0.034
Hip axis length (cm)	121.2 (6.3)	105.1 (6.7)	<0.001

^a^ *p* value for the difference between men and women

Key: *CSMI* cross sectional moment of inertia; *ED* endocortical diameter; *ACT* average cortical thickness; *PCD* profile centre distance; *CMP* centre of mass position

**Table 3:** Correlations between HSA variables and pQCT variables among HCS participants

	Men *r* (*p* value)	Women *r* (*p* value)
Association pQCT tibia width, 38 % slice and HSA variable		
Narrow neck width	0.35 (<0.001)	0.24 (<0.001)
Intertrochanter width	0.28 (<0.001)	0.32 (<0.001)
Femur shaft width	0.39 (<0.001)	0.45 (<0.001)
Association pQCT tibia endocortical diameter, 38 % slice and HSA variable		
Narrow neck endocortical diameter	0.26 (<0.001)	0.22 (<0.001)
Intertrochanter endocortical diameter	0.31 (<0.001)	0.34 (<0.001)
Femur shaft endocortical diameter	0.36 (<0.001)	0.47 (<0.001)
Association pQCT tibia cortical thickness, 38 % slice and HSA variable		
Narrow neck cortical thickness	0.39 (<0.001)	0.49 (<0.001)
Intertrochanter cortical thickness	0.46 (<0.001)	0.61 (<0.001)
Femur shaft cortical thickness	0.52 (<0.001)	0.63 (<0.001)
Association pQCT tibia polar strength strain index (ssi), 38 % slice and HSA variable		
Narrow neck section modulus	0.40 (<0.001)	0.37 (<0.001)
Intertrochanter section modulus	0.46 (<0.001)	0.48 (<0.001)
Femur shaft section modulus	0.54 (<0.001)	0.60 (<0.001)

## Discussion

To our knowledge, this is the first time that HSA and pQCT have been directly compared; here we have demonstrated strong correlations between two different methods of assessment of lower limb bone geometry and strength. We found strong relationships between tibial and femoral width; endocortical diameter; cortical thickness, and measures of bone strength in both men and women in their eighth decade.

Proximal femoral geometry is an independent determinant of hip fracture risk [7]. Whereas hip axis length is important in determining fracture risk, other measures of femoral geometry are also important contributors to strength. A previous large prospective cohort study of 7474 women looked at the predictive ability for future hip fracture of DXA-derived femur geometry parameters [9]. They found that hip fracture cases and controls significantly differ geometrically in several mechanically important ways that can be measured from DXA data. Hip fracture cases had larger neck-shaft angles, larger subperiosteal and estimated endosteal diameters, greater distances from lateral cortical margin to centre of mass, and higher estimated buckling ratios (*p* < 0.0001). Areal BMD, cross-sectional area, cross-sectional moment of inertia, section modulus, estimated cortical thickness and centroid position were all lower in hip fracture cases (*p* < 0.04).

In clinical studies, where pQCT measures have been related to fracture risk, an additional value of pQCT has been demonstrated. In a recent publication from the MrOS [15], 39 nontraumatic and nonvertebral fractures cases (60 % were hip, ankle/foot/toe or rib/chest/sternal fractures) were observed in a group of 1143 men aged 69 years or older, principal components analysis was used to identify 21 of 58 pQCT variables associated with incident fracture; of these variables, 18 still contributed to fracture risk, with AUC increasing from 0.73 to 0.80 with their inclusion. Of interest, tibial SSI was associated with incident fracture in this population, with a 9.6 % difference observed in mean values between men who did and did not fracture over follow-up.

We are not the first to report associations between different measures of bone geometry and strength. For example, Ohnauru et al. utilised hip computed tomography data from preoperative assessment of Japanese women undergoing hip joint replacement and compared these with HSA results based on DXA [16]. In that study the correlation between techniques was high for cortical thickness and section modulus in the both narrow neck and inintertrochanteric regions (*r* = 0.60–0.85). Although the correlation in the present study was numerically lower than reported by Ohnaru et al., this difference could be attributed to the difference in regions scanned using QCT. In our study, pQCT measurements of tibia were obtained, while they analysed the same regions between CT and DXA. In another study by Ramamurthi et al. where a sophisticated method to ensure that the same regions were analysed between CT and DXA the correlation between techniques for section modulus and width in the both narrow neck and inintertrochanteric regions was even stronger (*r* = 0.89–0.95) [17].

As expected, we found that bone size and strength as assessed by HSA were significantly higher in men than women (Table 2). Greater bone width in men was also associated with a greater endosteal circumference and average cortical thickness. These findings are likely to relate to sexual dimorphisms occurring during growth and the ageing process in later life, both of which lead to greater periosteal apposition in men than women [18, 19].

There are a number of strengths and weaknesses in this study. The main strengths of our study are that the sample investigated is generally representative of the UK population. However, there are also several limitations of this study. We used DXA images for assessment of proximal femoral geometry which, although not designed for this purpose, have been used in several validated studies of hip structure analysis. The areas imaged with DXA and pQCT are of course, different; namely hip and tibia, respectively, and images were not obtained contemporaneously, although we might expect this to obscure rather than strengthen any association. A large proportion of subjects seen at baseline were not included in the pQCT scan 4 years later. Selection bias is likely to be operating, and a healthy survivor effect may exist. However, our comparisons are internal justifying our decision to present these findings. Of note those individuals that took part in the later study differed from those that only attended the baseline clinic in that they were significantly younger, had a lower weight and BMI, and higher levels of physical activity. They were less likely to be a current or ex-smoker and to abstain from alcohol.

In conclusion, the results of this study show that there are strong correlations between two techniques to assess lower limb bone geometry and strength at the hip and tibia, respectively, namely HSA and pQCT. Each of these techniques has been independently associated with hip fracture risk in previous studies. Future work may now consider whether each technique offers an independent contribution to hip fracture prediction.
